# Fenestration and Bifurcation of the Internal Jugular Vein; Surprises During Head and Neck Surgery

**DOI:** 10.22038/ijorl.2025.83514.3810

**Published:** 2025

**Authors:** Vibha Singh, Arijit Jotdar, Annanya Soni, Rudra Prakash, Kushal Singh

**Affiliations:** 1 *Department of Otorhinolaryngology All India Institute of Medical Sciences, Raebareli, Uttar Pradesh, India.*; 2 *Department of Radiodiagnosis All India Institute of Medical Sciences, Raebareli, Uttar Pradesh, India.*

**Keywords:** Internal jugular vein, Fenestration, Bifurcation, Anatomical variation, Spinal accessory nerve

## Abstract

**Introduction:**

The internal jugular vein (IJV) is one of the major vessels in the neck and serves as an important landmark for surgeons during head and neck surgery. Anomalies of the IJV are rare and seldom encountered by the surgeons. However, a comprehensive knowledge of these variations is essential for better surgical dissection and to prevent intra-operative mishaps. The variations can be in the forms of bifurcation, trifurcation, duplication, fenestration and posterior tributaries of the IJV. Here we describe three cases of bifurcation and fenestration of the IJV that we encountered in our surgical practice.

**Case Report:**

In the first patient, we found an empty fenestration of the right internal jugular vein during a selective neck dissection for tongue carcinoma. The spinal accessory nerve was passing lateral to the IJV above the level of the fenestration. The second patient was operated for a left vagal schwannoma in the neck. During the surgery, we found a bifurcation of the left IJV, and the two tributaries fused just above the left omohyoid muscle. The third patient, a sixty-year-old lady also had a bifurcation of the left IJV. It was found during a modified radical neck dissection for carcinoma ex pleomorphic adenoma of the left parotid gland.

**Conclusion:**

An in-depth knowledge of the anomalies of the internal jugular vein and meticulous evaluation of the pre-operative imaging may help the surgeons in preventing any intra-operative catastrophe during head and neck surgery.

## Introduction

The internal jugular vein (IJV) is one of the most significant vascular landmarks for surgeons during head and neck surgery. As a continuation of the sigmoid sinus, this major vessel enters the neck through the jugular foramen and drains into the Subclavian vein to form the Brachiocephalic vein (1). It is an important landmark for the Spinal accessory nerve (SAN), Vagus nerve, Carotid artery and cervical lymph nodes (2). 

This large calibre vessel also serves as the route for central venous access and is hence important for anaesthetists and intensivists. Anatomical variations of the IJV are uncommon and are mostly reported by anaesthetists during imaging for central venous access (1). The Internal jugular vein receives both major and minor tributaries in the neck. Any major surgery in the neck involves the handling of this vessel. Therefore, an in-depth knowledge of its normal anatomy and associated variations is essential to avoid any untoward incident during surgical dissection. Any such anatomical variation of the IJV and adjacent vital structures especially the spinal accessory nerve can be diagnosed preoperatively by meticulous examination of the imaging studies including CT scans and MRI scans, which are routinely performed for any head and neck surgery. However, due to its rarity, the variations of the IJV are sometimes overlooked and remain undetected preoperatively. 

## Case Report

### Case 1:

A 48-year-old lady presented in the department of Otorhinolaryngology with a non-healing ulcer over the right lateral border of the tongue. Biopsy from the lesion yielded a well-differentiated squamous cell carcinoma. The patient was staged as cT_2_N_1_M_0_ after all necessary investigations. She underwent Wide local excision with selective neck dissection (I-IV) with primary repair. 

**Fig 1:**
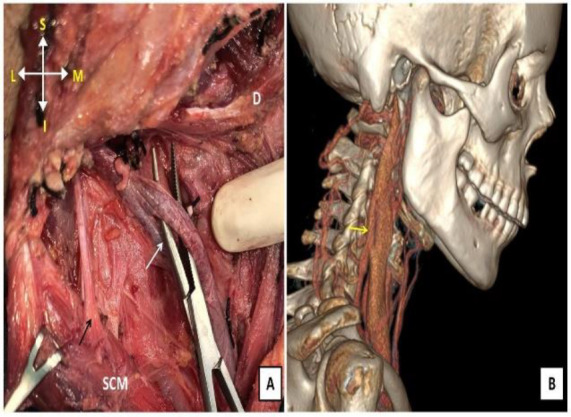
A. Empty fenestration of the right internal jugular vein (white arrow). The spinal accessory nerve is passing lateral to the IJV above the level of the fenestration (black arrow). The sternocleido- mastoid (SCM) and digastric (D) muscles are also seen in the surgical field. B. Reconstructed virtual CT scan of the patient showing the empty fenestration of the right internal jugular vein (yellow arrow).

During neck dissection, we found that the right internal jugular vein split into two branches just below the level of the hyoid bone. Both the branches fused again to form a single trunk of the IJV at the level of the cricoid cartilage. No significant neurovascular structure was found passing between the branches making it an empty fenestration (Figure 1). The spinal accessory nerve was found separately above the level of fenestration. Fibrofatty tissue was gently dissected from both the branches and neck dissection was completed. The intraoperative period was uneventful with no injury to the internal jugular vein and spinal accessory nerve. Postoperatively virtual CT scan was reviewed which confirmed an empty fenestration of the right internal jugular vein. 

### Case 2:

A 19-year-old gentleman presented with a gradually progressive swelling over the left side of the neck for the last one year. Fine needle aspiration cytology pointed towards a spindle cell neoplasm. MRI scan was suggestive of a vagal schwannoma. 

The patient was taken up for trans-cervical excision of vagal schwannoma under general anaesthesia. Intraoperatively, the left common carotid artery and the left internal jugular vein were found to be pushed medially by the tumour. 

**Fig 2:**
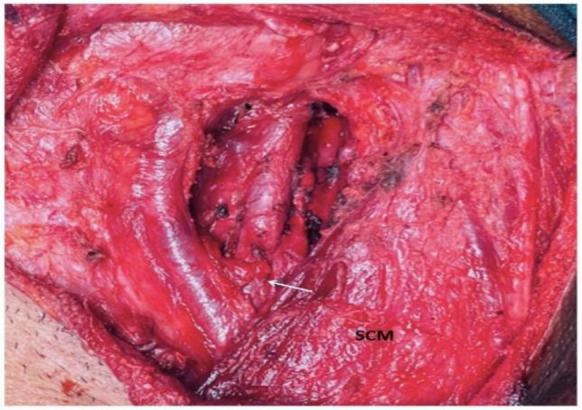
Bifurcation of the left internal jugular vein (white arrow). Both the tributaries fused above the level of the omohyoid forming the main trunk of the internal jugular vein.

Another major vessel was found to be running vertically over the tumour, which could be traced back up to the skull base. On incising the carotid sheath, we found a bifurcation of the left internal jugular vein just above the level of the cricoid cartilage (Figure 2). The tumour was carefully separated from the internal jugular vein, thereby preserving both of its branches. 

### Case 3:

**Fig 3:**
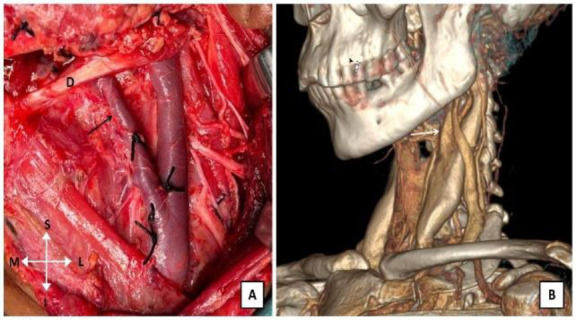
A. Bifurcation of the left internal jugular vein (black arrow). Both the branches fuse above the level of the omohyoid muscle to form the main trunk of the IJV. The digastric muscle (D) is seen in the surgical field. B. Reconstructed virtual CT scan of the patient showing bifurcation of the left internal jugular vein (white arrow).

A 60-year-old lady presented with a gradually progressive swelling of the left parotid region for the last one year. A fine needle aspiration cytology from the swelling was suggestive of carcinoma ex pleomorphic adenoma of the left parotid gland. The patient was staged cT_3_N_0_M_0_ after all necessary investigations. She underwent left total conservative parotidectomy with left-sided modified radical neck dissection (level I–V). During the dissection of cervical lymph nodes, two branches of the left internal jugular vein were found arising deep into the digastric muscle. Both the branches continued separately up to the level of the cricoid cartilage and then fused above the omohyoid muscle confirming a bifurcation of the left internal jugular vein (Figure 3). This was further established by a careful examination of the virtual CT scan. 

## Discussion

Anomalies of the internal jugular vein are rare and often remain undetected pre-operatively during head and neck surgery. This may be in the forms of bifurcation, duplication, fenestration, trifurcation and posterior tributary of the internal jugular vein (1).

 Mumtaz S et al in a literature review of 27 articles consisting of 1197 cases, reported only 40 cases of anatomical variations of the internal jugular vein. Among these cases, bifurcation was found only in four cases, and fenestration in sixteen cases. Although rare, the prevalence of the IJV anomalies is variable in different studies (Table 1). 

**Fig 4:**
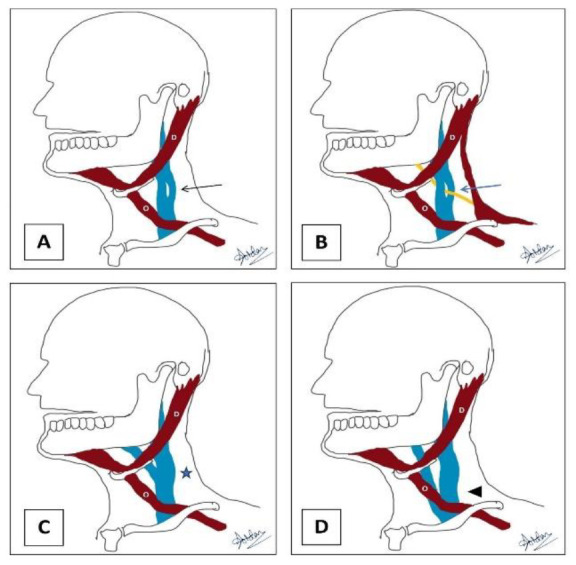
Schematic diagram representing the anatomical variations of the internal jugular vein; A. Empty fenestration of the internal jugular vein (black arrow). B. Fenestration in the internal jugular vein transmitting the spinal accessory nerve through it (blue arrow). C. Bifurcation of the IJV above the level of the omohyoid muscle (O), marked by a blue star. D. Duplication of the IJV below the level of the omohyoid muscle, marked by a black arrowhead.

Some ambiguity exists among the head and neck surgeons regarding the terms ‘bifurcation’ and ‘duplication’ of the IJV. Some authors suggest the term ‘bifurcation’ when the IJV splits above the level of the omohyoid muscle (1). In our case series, the second and third cases had a bifurcation of the internal jugular vein as both branches fused above the level of the omohyoid muscle (Figure 4). The relation of the spinal accessory nerve (SAN) with the internal jugular vein needs special mention in this context. In most of the cases, the spinal accessory nerve passes lateral to the IJV (56 – 90%) followed by medial to the IJV (10 – 44%) (3,4). However, in extremely rare cases, the SAN may pass through a fenestration in the IJV with only a few reported cases in the literature (5-7). Empty fenestration of the internal jugular vein is even rarer. In such cases, no neurovascular bundle or important structure passes through the fenestration of the IJV (Figure 4). Sometimes the ipsilateral internal carotid artery may coil through a fenestration in the IJV (8). In our case series, the first patient had an empty fenestration in the IJV with the spinal accessory nerve passing lateral to it.

The internal jugular vein is one of the largest vessels encountered during head and neck surgeries. Anomalies of the IJV are usually incidental findings during neck explorations. Non-meticulous dissection intraoperatively in such cases can lead to injury to the IJV as well as to the spinal accessory nerve. This can result in significant post-operative morbidity to the patient. It can also cause a blood-filled operative field thereby increasing the intraoperative time. 

These anomalies can be identified pre-operatively on careful examination of the imaging. The surgeon should be aware and vigilant about these anatomical variations of the IJV that can improve the surgical dissection and the overall postoperative outcome. It can also be helpful in radical neck dissection where the IJV needs to be sacrificed. In this article, we reported three cases of rare anatomical variations of the internal jugular vein.

## Conclusion

Anatomical variations of the internal jugular vein are rare and often found incidentally during head and neck surgeries. A meticulous evaluation of the pre-operative imaging studies may help the surgeons to be vigilant and cautious during dissection around the internal jugular vein in such situations. 

**Table 1:** Prevalence of anatomical variations of the IJV in world literature

Sl No	Author	Study population	Reported cases of anatomical variations	Types of anatomical variations	Prevalence
**1**	Prades et al (4)	750	3	Duplication	0.4%
**2**	Wang X et al (9)	221	2	Fenestration & duplication	0.9%
**3**	Hashimoto et al (10)	123	4	Fenestration	3.3%
**4**	Contrera et al (11)	295	3	Fenestration, duplication and bifurcation	1%
